# Pathogenic variants in the paired-related homeobox 1 gene (*PRRX1*) cause craniosynostosis with incomplete penetrance

**DOI:** 10.1016/j.gim.2023.100883

**Published:** 2023-09

**Authors:** Rebecca S. Tooze, Kerry A. Miller, Sigrid M.A. Swagemakers, Eduardo Calpena, Simon J. McGowan, Odile Boute, Corinne Collet, David Johnson, Fanny Laffargue, Nicole de Leeuw, Jenny V. Morton, Peter Noons, Charlotte W. Ockeloen, Julie M. Phipps, Tiong Yang Tan, Andrew T. Timberlake, Clemence Vanlerberghe, Steven A. Wall, Astrid Weber, Louise C. Wilson, Elaine H. Zackai, Irene M.J. Mathijssen, Stephen R.F. Twigg, Andrew O.M. Wilkie

**Affiliations:** 1 Clinical Genetics Group, MRC Weatherall Institute of Molecular Medicine, University of Oxford, Oxford, United Kingdom; 2 Department of Pathology & Clinical Bioinformatics, Erasmus University Medical Center Rotterdam, Rotterdam, The Netherlands; 3 Centre for Computational Biology, MRC Weatherall Institute of Molecular Medicine, University of Oxford, Oxford, United Kingdom; 4 Univ. Lille, CHU Lille, ULR 7364 – RADEME – Maladies Rares du Développement Embryonnaire et du Métabolisme, Clinique de Génétique, Lille, France; 5 Genetics Department, Robert Debré University Hospital, APHP, Paris, France; 6 Craniofacial Unit, Oxford University Hospitals NHS Foundation Trust, Oxford, United Kingdom; 7 Clinical Genetics Service and Reference Centre for Rare Developmental Abnormalities and Intellectual Disabilities, University Hospital of Clermont-Ferrand, Clermont-Ferrand, France; 8 Department of Human Genetics, Radboud University Medical Center, Nijmegen, The Netherlands; 9 West Midlands Regional Clinical Genetics Service and Birmingham Health Partners, Birmingham Women’s and Children’s Hospitals NHS Foundation Trust, Birmingham, United Kingdom; 10 Department of Craniofacial Surgery, Birmingham Children's Hospital NHS Foundation Trust, Birmingham, United Kingdom; 11 Oxford Centre for Genomic Medicine, Oxford University Hospitals NHS Foundation Trust, Oxford, United Kingdom; 12 Victorian Clinical Genetics Services, Murdoch Children’s Research Institute, Royal Children’s Hospital, Melbourne, Australia; 13 Department of Paediatrics, University of Melbourne, Melbourne, VIC, Australia; 14 Hansjörg Wyss Department of Plastic Surgery, NYU Langone Medical Center, New York, NY; 15 Liverpool Centre for Genomic Medicine, Liverpool Women’s NHS Foundation Trust, Liverpool, United Kingdom; 16 North East Thames Regional Genetics Service, Great Ormond Street Hospital for Children NHS Foundation Trust, London, United Kingdom; 17 Clinical Genetics Center, Division of Human Genetics, Children's Hospital of Philadelphia, Philadelphia, PA; 18 Department of Plastic and Reconstructive Surgery and Hand Surgery, Erasmus Medical Centre, University Medical Centre Rotterdam, Rotterdam, The Netherlands

**Keywords:** Craniosynostosis, Homeodomain, Nuclear localization, Paired-related homeobox, PRRX1/PRX1

## Abstract

**Purpose:**

Studies have previously implicated PRRX1 in craniofacial development, including demonstration of murine *Prrx1* expression in the preosteogenic cells of the cranial sutures. We investigated the role of heterozygous missense and loss-of-function (LoF) variants in *PRRX1* associated with craniosynostosis.

**Methods:**

Trio-based genome, exome, or targeted sequencing were used to screen *PRRX1* in patients with craniosynostosis; immunofluorescence analyses were used to assess nuclear localization of wild-type and mutant proteins.

**Results:**

Genome sequencing identified 2 of 9 sporadically affected individuals with syndromic/multisuture craniosynostosis, who were heterozygous for rare/undescribed variants in *PRRX1*. Exome or targeted sequencing of *PRRX1* revealed a further 9 of 1449 patients with craniosynostosis harboring deletions or rare heterozygous variants within the homeodomain. By collaboration, 7 additional individuals (4 families) were identified with putatively pathogenic *PRRX1* variants. Immunofluorescence analyses showed that missense variants within the PRRX1 homeodomain cause abnormal nuclear localization. Of patients with variants considered likely pathogenic, bicoronal or other multisuture synostosis was present in 11 of 17 cases (65%). Pathogenic variants were inherited from unaffected relatives in many instances, yielding a 12.5% penetrance estimate for craniosynostosis.

**Conclusion:**

This work supports a key role for PRRX1 in cranial suture development and shows that haploinsufficiency of *PRRX1* is a relatively frequent cause of craniosynostosis.

## Introduction

Craniosynostosis, the premature fusion of one or more of the cranial sutures of the skull, occurs with a prevalence of approximately 1 in 2000 births.^1^^,^^2^ It is characterized by the combination of sutures fused (sagittal, metopic, coronal, and lambdoid) and the presence of additional abnormal physical or developmental features, consistent with a syndrome. An underlying genetic cause can be found in approximately one quarter of affected individuals by identification of pathogenic variants in >50 genes (with *EFNB1*, *ERF*, *FGFR2*, *FGFR3*, *SMAD6*, *TCF12*, and *TWIST1* most frequently implicated) or diverse chromosomal abnormalities.^3^, ^4^, ^5^ Many of these genes play key roles in cranial suture biology, from developmental patterning to the maintenance of stem cell/progenitor proliferation-differentiation balance within the suture during calvarial expansion.^3^ Here, we present evidence implicating pathogenic variants of *PRRX1* in craniosynostosis.

**Figure 1:**
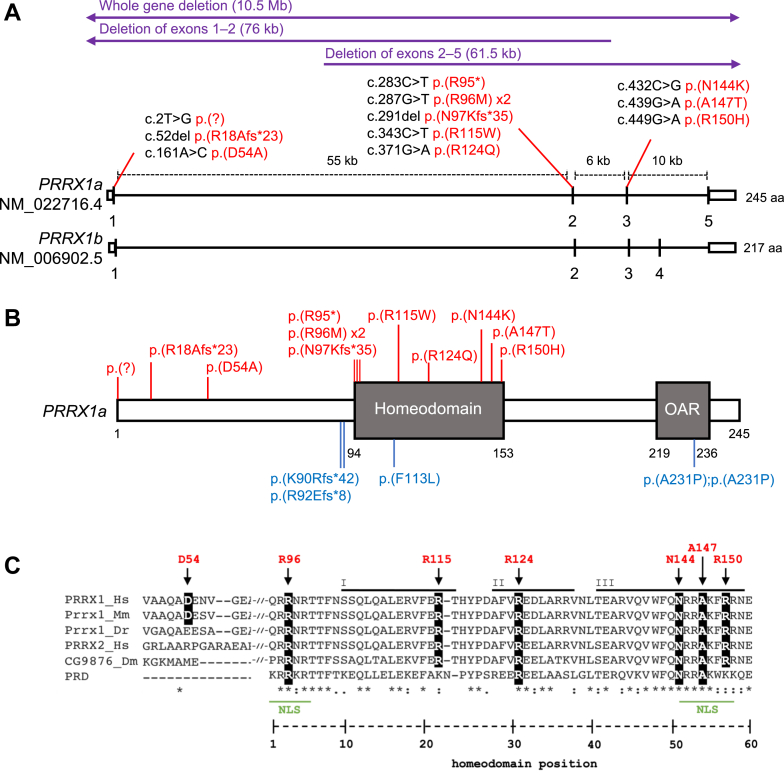
**Structure, conservation, and variants in *PRRX1***. A. A schematic of the exon structure (exons 1-5) of human *PRRX1a* (245 aa) and *PRRX1b* (217 aa). Alternative splicing of the final exon results in an OAR (otp, aristaless, rax) domain present in PRRX1a, which is absent in PRRX1b. Variants identified in patients with craniosynostosis are highlighted in red with the purple lines above indicating the 3 deletions identified in independent patients. Arrows indicate that the deletion extends beyond the *PRRX1* gene. B. A schematic representation of the PRRX1a protein (P54821) showing the position of the homeodomain and the OAR domain. Variants identified in this study are highlighted in red, whereas variants reported in patients with agnathia-otocephaly are shown in blue. The only homozygous variant reported is p.(A231P) identified in a patient with agnathia-otocephaly. C. Conservation of the amino acids surrounding D54 (left) and the 60 amino acids of the homeodomain (right). Sequences correspond to human PRRX1a and PRRX2 (PRRX1_Hs and PRRX2_Hs, respectively), mouse (Prrx1_Mm), zebrafish (Prrx1_Dr), and Drosophila (CG9876_Dm), and the consensus sequence for the PRD class of homeodomain-containing proteins. The arrows indicate the position of the missense substitutions identified in this study. The 3 alpha helices of the homeodomain are also highlighted (I, II, and III). Two predicted nuclear localization sequences (NLS) are annotated in green. Asterisks (∗) represent complete conservation across all alignments, a colon (:) represents aligned residues with similar biochemical properties, and the period (.) denotes conservation between groups with weakly similar properties. aa, amino acid.

The mammalian paired-related homeobox family comprises 2 genes, *PRRX1* and *PRRX2*, which are classified within the PRD class of homeobox transcription factors.^6^ The orthologous genes in mice (*Prrx1* and *Prrx2*) were previously named *MHox*/*Prx1* and *Prx2*, respectively. *PRRX1* comprises 5 exons; alternative splicing of exon 4 generates 2 distinct protein isoforms, *PRRX1a* (NM_022716.4; 245 amino acids [aa] in humans) and *PRRX1b* (NM_006902.5; 217 aa) (Figure 1A). *PRRX1a* and *PRRX2* (NM_016307.4) (which is not subject to alternative splicing) contain a highly conserved C-terminal OAR (otp, aristaless, and rax) domain (Figure 1B), which is absent in *PRRX1b*.^7^

*Prrx1* has been shown to be widely expressed within the mouse coronal suture at embryonic day (E) 15.5 and is a marker of stem cells in the postnatal sutural mesenchyme.^8^, ^9^, ^10^ Postnatal skeletal stem cells expressing *Prrx1* reside exclusively within the calvarial suture, respond to wingless-related integration site (WNT) signaling by differentiating into osteoblasts, and are able to regenerate bone upon heterotopic transplantation.^11^ *Prrx1* and *Prrx2* exhibit overlapping expression patterns in undifferentiated mesenchyme of the head, limb buds, axial mesoderm, and branchial arches, although there are differences, particularly in the heart and brain.^12^^,^^13^ Analysis of mouse null mutants demonstrated partially redundant roles of *Prrx1* and *Prrx2* in craniofacial development. Although *Prrx1* heterozygotes were normal, *Prrx1* null mice died at birth with cleft palate, defects of multiple bones of the face and lateral skull, and absent supraoccipital bone; additional anomalies were present in the limbs (short, thickened endochondral bones) and vertebrae (incomplete vertebral arches).^14^ Although *Prrx2* null mice were viable and fertile (including when combined with *Prrx1*^*+/−*^), *Prrx1*^*−/−*^;*Prrx2*^*−/−*^ double mutants exhibited a marked exacerbation of the *Prrx1*^*−/−*^ phenotype, including novel features, indicating dosage sensitivity and functional redundancy.^13^^,^^15^

Influenced by the earlier mouse work, *PRRX1* (located at human chromosome 1q24.2) was previously tested as a candidate disease gene in infants with agnathia-otocephaly complex (MIM 202650), a severe craniofacial malformation in which reduction or absence of the mandible is associated with microstomia, hypo- or a-glossia and ventromedial auricular malposition or fusion. After the identification of a heterozygous variant in *PRRX1* (NM_022716.4:c.337T>C; p.(Phe113Leu) [incorrectly documented as p.(Phe113Ser) in the original report]),^16^ 3 further reports of homozygous (NM_022716.4:c.691G>C; p.(Ala231Pro))^17^ or heterozygous variants have been published, the latter comprising 2 de novo frameshift variants: NM_022716.4:c.266_269dup; p.(Arg92Glufs∗8), incorrectly reported as p.(Arg92Glufs∗98),^18^ and NM_022716.4:c.269del; p.(Lys90Argfs∗42), incorrectly reported as NM_022716.4:c.267del; p.(Lys90Argfs∗131).^19^

Here, we used genome and exome sequencing and targeted resequencing to demonstrate that rare *PRRX1* variants are enriched in individuals with craniosynostosis. Of the 17 individuals with deleterious variants, 3 were present within a prospective UK 18-year birth cohort of 981 affected individuals (unpublished data), indicating that *PRRX1* pathogenic variants are a relatively frequent (0.3% overall) cause of craniosynostosis. These findings accord with recent observations that *Prrx1* is expressed in the major cranial sutures, identifying a subset of cells with characteristics of osteoprogenitors.^8^^,^^9^^,^^11^^,^^20^

## Materials and Methods

### Genome and exome sequencing

Genome sequencing (GS) of 9 trios comprising a sporadically affected child with molecularly undiagnosed syndromic/multisuture craniosynostosis was performed by Complete Genomics (Mountain View) following the protocols from Drmanac et al^21^; de novo variant analysis was performed as previously described.^22^^,^^23^ Variants were annotated using GRCh37/hg19 and dbSNP build 130. Variants identified in *PRRX1* were confirmed by dideoxy sequencing of genomic polymerase chain reaction (PCR) amplification products (Supplemental Figure 1B). Exome sequencing of 520 unrelated probands (Yale WES Cohort, Supplemental Table 1) with genetically undiagnosed craniosynostosis was previously described.^24^

### Targeted resequencing of *PRRX1*

A combination of PCR and high-throughput sequencing was used to screen samples from 388 patients with craniosynostosis without a molecular diagnosis, for variants in *PRRX1*. Primers (Supplemental Table 2) were designed to amplify all coding regions of *PRRX1* with the addition of CS1 (5′-ACACTGACGACATGGTTCTACA-3′) and CS2 (5′-TACGGTAGCAGAGACTTGGTCT-3′) adaptor sequences included on the 5′ ends of all target-specific forward and reverse primers, respectively (details in Supplemental Methods). An additional 541 samples were screened for pathogenic variants in *PRRX1* using Integrated DNA Technologies’ hybridization and capture protocol (details in Supplemental Methods). Probes were designed to ensure that all coding regions of *PRRX1* were captured by at least 2 probes (Supplemental Table 3). Sequencing data were analyzed using amplimap software^25^ (including mapping, coverage analysis, and variant calling), and variants were filtered on the basis of rarity (allele frequency in gnomAD [v2.1.1]^26^ below 0.000045),^27^ Combined Annotation Dependent Depletion score (≥20 or not reported), and likely consequence (missense or more damaging).

The coverage of *PRRX1* was assessed in a subset of patients (*n* = 479) included in the targeted sequencing analysis. These samples were selected because they displayed low variability in normalized mean coverage (see Supplemental Methods for further details). Total coverage for nonoverlapping probe regions (including 20 regions covering all exons) was normalized by the mean coverage for all genes analyzed (*n* = 41) for each sample, and by the average coverage for all samples for each probe region. Each sample was analyzed for deletions or duplications, considering ≥2 consecutive probes displaying a normalized coverage below 0.6 (heterozygous deletion) or above 1.4 (heterozygous duplication).

### Isolation of the breakpoints in patients with deletions in *PRRX1*

A 25 ng sample of genomic DNA was mixed with 12.5 μL of 2× Q5 High-Fidelity Master Mix (NEB, M0492S), 1.25 μL of each forward and reverse primer (Supplemental Table 4) (10 mM), and up to 25 μL total volume of nuclease-free water. The sample was amplified in a thermocycler at 98 °C for 30 seconds, followed by 35 cycles of 95 °C for 10 seconds, 66 °C for 30 seconds, and 72 °C for 30 seconds, and a final extension of 72 °C for 2 minutes. A control primer pair amplifying a region of *SYNJ2* (SYNJ2_FW: 5′-CCACTGTGTTGAGCTGATGA-3′ and SYNJ2_RV: 5′-CAACGGGAAATGCTGCAAAG-3′) was used to confirm the presence of a band in a healthy control sample (Supplemental Figure 2A). The PCR product corresponding to the *PRRX1*-deletion was analyzed by dideoxy sequencing to establish the breakpoints (Supplemental Figure 2B and C).

### Plasmid construction

Constructs containing murine *Prrx1* complementary DNA (cDNA) were originally obtained as a gift from Michael Kern,^7^ and missense variants were derived in these constructs using site-directed mutagenesis (primers in Supplemental Table 5). Subsequently, *Prrx1* was amplified with primers containing EcoRI and HindIII restriction sites at the 5′ and 3′ ends, respectively (Supplemental Table 6), before subcloning into a hemagglutinin (HA) and FLAG-tagged (N-terminal to *Prrx1*) vector obtained from Addgene (FLAG-HA-pcDNA3.1, #52535). All constructs were confirmed by dideoxy sequencing (Supplemental Figure 3). Further experimental details are provided in the Supplemental Methods.

### Immunofluorescence

COS7 cells were seeded onto coverslips to reach 60% confluency after 24 hours in a 6-well plate, before transfection with 0.5 μg of plasmid and 3 μL of Lipofectamine-2000 (ThermoFisher Scientific). After 18 hours, cells were fixed using paraformaldehyde (4%, pH 7.4) and permeabilized using 0.2% Triton X-100, before blocking with 2% bovine serum albumin. Anti-HA (HA-Tag [C29F4], Rabbit mAb #3724, Cell Signaling) was diluted 1:100 in SignalBoost Immunoreaction Enhancer Solution 1 (Calbiochem KP31812) and added to coverslips to incubate for 1 hour at room temperature. Coverslips were washed with 1× phosphate-buffered saline (PBS) before addition of secondary antibody (Alexa Fluor 488 donkey anti-rabbit IgG [Life Technologies, #A-21206]), diluted 1:500 in SignalBoost Immunoreaction Enhancer Solution 2 (Calbiochem, KP31855). Cells were incubated with secondary antibody for 45 minutes at room temperature and counterstained with Alexa Fluor 647 Phalloidin diluted 1:20 in 1× PBS (Cell Signaling, #8940) for 15 minutes, followed by staining with 4′,6-diamidino-2-phenylindole (DAPI, 2 mg/mL) diluted 1:2000 in 1× PBS for 10 minutes. Coverslips were washed and mounted on slides using VECTASHIELD Antifade Mounting Medium H-1000 (Vector Laboratories) and imaged using Zeiss 880 Inverted Confocal (Wolfson Imaging Centre, WIMM) and ScanR microscopes (Micron Imaging Facility, Department of Biochemistry, University of Oxford). The ScanR microscope imaged 25 random sections of each slide (in technical triplicates) and cells were counted for distribution of protein within the nucleus or cytoplasm, blinded to the transfected construct used. A minimum of 698 cells were counted for each variant.

## Results

### Identification of rare heterozygous variants of *PRRX1* in craniosynostosis

Initial analysis of GS data from 9 parent-child trios with previously undiagnosed syndromic/multisuture craniosynostosis (Supplemental Table 1) revealed a de novo single-nucleotide deletion of *PRRX1* (NM_022716.4:c.52del, predicting a frameshift p.(Arg18Alafs∗23)), in a child (family 2, II-1; Table 1) presenting with bicoronal synostosis. Given the previous evidence implicating PRRX1 function in craniofacial development,^14^^,^^15^ we examined the GS data for additional variants. This revealed a heterozygous *PRRX1* variant, NM_022716.4:c.449G>A; p.(Arg150His), in individual II-3 from family 12 (Table 1), which had not previously been prioritized because it was inherited from the apparently unaffected father. This variant encoded an arginine to histidine substitution at the 57th position of the highly conserved 60 aa homeodomain. No nonsynonymous variants at Arg150 have been identified in >250,000 *PRRX1* alleles (gnomAD v2.1.1),^26^ and substitutions of the equivalent arginine have been reported as pathogenic in other homeodomain proteins, for example, SHOX, in which the equivalent substitution was shown to abolish nuclear import through disruption of the nuclear localization signal.^28^^,^^29^

**Table 1:** Rare heterozygous *PRRX1* (NM_022716.4) variants identified in this study

Family No.	Individuals With Variants^a^	Suture Fusion^c^	Syndromic/Nonsyndromic^d^	Exon(s) No.	Chromosome and Position (GRCh38)	cDNA Change	Amino Acid Change	CADD Score	gnomAD Prevalence (v2.1.1)	De Novo?	% Nuclear Localization, Mean ± SD
1	I-1, II-1^a^^,^^b^	S+LL	ns	1	chr1:170664220	c.2T>G	p.(?)	24.3	0		–
2	II-1^a^^,^^b^	BC	ns	1	chr1:170664268	c.52delC	p.(Arg18Alafs∗23)	–	0	Y	–
3	I-2, II-3^a^^,^^b^	Me	s	1	chr1:170664379	c.161A>C	p.(Asp54Ala)	23.3	0		78.7 ± 4.1
4	I-1, II-1^a^^,^^b^	S+RC	ns	2	chr1:170719767	c.283C>T	p.(Arg95∗)	36	0		–
5	I-2, II-2, II-3, III-2^a^^,^^b^	S	ns	2	chr1:170719771	c.287G>T	p.(Arg96Met)	29.5	0		39.1 ± 1.8
6	I-2, II-2, III-2^a^^,^^b^	RC	ns	2	chr1:170719771	c.287G>T	p.(Arg96Met)	29.5	0		39.1 ± 1.8
7	II-3, III-1^a^^,^^b^, III-2^b^	BC; P	s; ns	2	chr1:170719774	c.291delT	p.(Asn97Lysfs∗35)	–	0		–
8	II-2, II-3, III-1^a^^,^^b^, III-3^b^	S; S	s; s	2	chr1:170719827	c.343C>T	p.(Arg115Trp)	23.4	1.06 × 10^−5^		50.4 ± 6.6
9	II-1, III-1^a^^,^^b^	RL	ns	2	chr1:170719855	c.371G>A	p.(Arg124Gln)	31	0	Y (in II-1)	49.0 ± 0.4
10	I-2^b^, II-2, III-1^a^^,^^b^, III-2	BC; BC	s; s	3	chr1:170726234	c.432C>G	p.(Asn144Lys)	25.2	0		49.5 ± 3.7
11	I-2, II-3^a^^,^^b^	S+BC	ns	3	chr1:170726241	c.439G>A	p.(Ala147Thr)	28.9	0		56.3 ± 5.1
12	I-1, II-3^a^^,^^b^	S+BC	s	3	chr1:170726251	c.449G>A	p.(Arg150His)	31	0		49.3 ± 8.4
13	II-1^a^^,^^b^	Mu	ns	2-5	chr1:170692716-170754397	–	61.5 kb deletion	–	0	Y	–
14	I-2, II-2^a^^,^^b^	LC	ns	1-2	chr1:170649419-170725333	–	76 kb deletion	–	0		–
15	II-1^a^^,^^b^	BC	s	1-5	–	–	10.5 Mb deletion	–	0		–

CADD, Combined Annotation Dependent Depletion; cDNA, complementary DNA.

a Index patient.

b Individuals with craniosynostosis.

c BC refers to bicoronal, LC refers to left coronal, Me refers to metopic; Mu refers to multi-suture synostosis, P refers to pansynostosis, RC refers to right coronal; RL refers to right lambdoid, and S refers to sagittal.

d ns refers to nonsyndromic, and s refers to syndromic.

Based on these preliminary findings, we interrogated a cohort of patients with craniosynostosis (largely with sagittal or metopic fusion) previously analyzed by exome sequencing (*n* = 520)^24^ and resequenced a separate cohort of patients with craniosynostosis (considering a broader range of suture fusion phenotypes) who had not previously received a formal genetic diagnosis (*n* = 929) (Table 2, Supplemental Table 1). This revealed 7 further unrelated subjects heterozygous for rare, predicted pathogenic variants in *PRRX1*. These comprised a loss of the start codon (NM_022716.4:c.2T>G; p.(?), family 1), a nonsense variant (NM_022716.4:c.283C>T; p.(Arg95∗), family 4), and 4 amino acid substitutions within the homeodomain (arginine to methionine at the third homeodomain residue [NM_022716.4:c.287G>T; p.(Arg96Met), families 5 and 6], arginine to glutamine at the 31st position of the homeodomain [NM_022716.4:c.371G>A; p.(Arg124Gln), family 9], and alanine to threonine at the 54th homeodomain residue [NM_022716.4:c.439G>A; p.(Ala147Thr), family 11]). Additionally, a missense variant at a highly conserved residue N-terminal to the homeodomain was identified (NM_022716.4:c.161A>C; p.(Asp54Ala), Family 3) (Table 1). To provide a comparator, we examined data from the gnomAD database (v2.1.1) and UK Biobank.^30^ In approximately 250,000 alleles in gnomAD, there are 4 LoF alleles and 34 missense substitutions (excluding a known benign substitution, p.(Ser104Gly)) that potentially disrupt the homeodomain. In approximately 735,000 alleles in the UK Biobank, there are 3 LoF variants and 110 missense variants (excluding p.(Ser104Gly)) predicted to disrupt the homeodomain. This indicates a significant over-representation (16-fold; Fisher exact test: *P* < .000001) in the resequencing study (2 LoF and 5 homeodomain missense variants in 2898 alleles) compared with gnomAD and the UK Biobank, consistent with a causal contribution in the affected individuals.

**Table 2:** Subjects with craniosynostosis analyzed for rare, deleterious *PRRX1* variants in 3 screens^a^

Suture Fused	Nonsyndromic		Syndromic		Combined	
	Total	PRRX1 Positive	Total	PRRX1 Positive	Total	PRRX1 Positive
Metopic	321		46	1	367	1 (0.27%)
Sagittal	611	1	57		668	1 (0.15%)
Unilateral coronal	180	2	24		204	2 (0.98%)
Bilateral coronal	33	1	11	1	44	2 (4.55%)
Uni- or bilateral lambdoid	33	1	4		37	1 (2.70%)
Other multisuture	70	3	46	1	116	4 (3.45%)
Suture not specified			19		19	0 (0.00%)
Combined	1248	8	207	3	1455	11 (0.76%)

a See Supplemental Table 1 for further details.

Given the evidence that small nucleotide variants of *PRRX1* are enriched in craniosynostosis, we used 2 approaches to identify further index cases with pathogenic *PRRX1* variants. First, we developed a robust method to screen for copy number changes of *PRRX1* in the targeted capture data (*n* = 479). This identified 2 individuals, 1 heterozygous for a partial *PRRX1* deletion including exons 1 and 2 only (family 14) and the other with a whole gene deletion (family 15; Supplemental Figure 4A and B); both deletions were independently confirmed using array-based methodology, and the breakpoints of the partial deletion (which extended ∼76 kb) were isolated using PCR amplification (Supplemental Figure 2). Second, through collaboration with the community of craniofacial geneticists, we identified 4 families with potentially pathogenic heterozygous variants, comprising 3 additional variants within the homeodomain (families 7, 8, and 10; Table 1) and a 61.5 kb deletion, including exons 2 to 5 (family 13).

### PRRX1 missense substitutions affect nuclear localization

**Figure 2:**
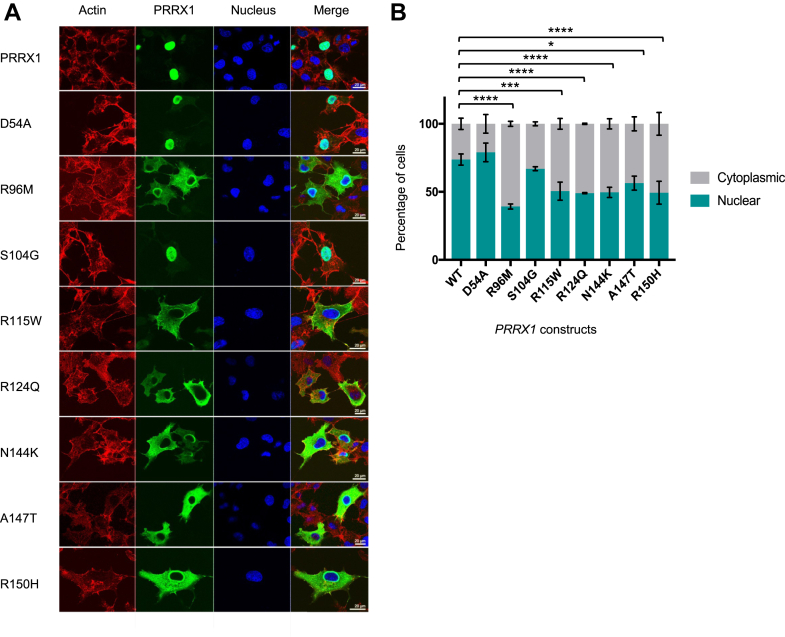
**Immunofluorescence analysis of PRRX1 homeodomain missense substitutions.** A. Confocal microscopy was used to analyze the distribution of PRRX1 in the nucleus or cytoplasm of COS7 cells transfected with plasmids containing wild-type *Prrx1* or mutant (D54A, R96M, S104G, R115W, R124Q, N144K, A147T, and R150H). From left to right images show cytoplasmic staining of actin using Phallodin-647 (red), staining of HA-tagged PRRX1 (green), nuclear staining using DAPI (blue), and an overlay of the 3 channels. Images are representative of the major phenotype assessed over 3 technical replicates. B. Results were quantified using a ScanR microscope that randomly took images of 25 sections of each slide (previously analyzed using confocal microscopy). Blinded cell counts were scored as either cytoplasmic or nuclear considering a total of >698 cells per construct. Error bars represent ± SD. A two-way analysis of variance (with Bonferroni correction) was used to assess the difference between nuclear distribution of cells expressing the wild-type and mutant proteins (*P* values: ∗.0156, ∗∗∗ <.005, ∗∗∗∗ ≤.0001). WT, wild-type.

To investigate the functional consequences of missense variation, we transfected *Prrx1* constructs into COS7 cells and undertook immunofluorescence analysis to establish if any of the variants caused abnormal nuclear localization (Figure 2A). We considered the wild-type protein (PRRX1), a homeodomain variant with an allele frequency of 0.0011 in gnomAD v2.1.1 that is classified as benign (p.(Ser104Gly)), the 7 missense variants identified in patients with craniosynostosis, and a heterozygous missense variant in the homeodomain (p.(Phe113Leu)) reported in an individual with agnathia-otocephaly.^16^ Images were generated by confocal microscopy and analyzed using a ScanR microscope whereby cells were classified as having either a cytoplasmic or nuclear distribution of PRRX1 (Figure 2A) (698-1187 cells counted for each variant). After transfection of the wild-type *PRRX1* construct, the encoded protein localized within the nucleus in 74% of cells counted (averaged across 3 repeats) (Figure 2B); quantitatively similar results were obtained for the p.(Ser104Gly) polymorphism (67%) and the p.(Asp54Ala) variant (79%; this being the only missense substitution outside the homeodomain). In contrast, all 6 of the craniosynostosis-associated homeodomain missense substitutions demonstrated abnormal localization of PRRX1, with the protein localizing to the cytoplasm in the majority of cells. The most severe quantitative defect was obtained for cells transfected with the p.(Arg96Met) variant, for which PRRX1 localized to the cytoplasm in 61% of cells (a 35% reduction in nuclear localization compared with wild-type PRRX1); the other homeodomain missense substitutions displayed a 17% to 25% reduction in cells displaying nuclear localization compared with wild type. Interestingly, for the agnathia-otocephaly variant (p.(Phe113Leu)),^16^ 82% of cells displayed a nuclear phenotype (similar to the wild-type protein), suggesting a different pathogenic mechanism(s) for this variant (Supplemental Figure 5).

### Clinical phenotype of individuals with heterozygous *PRRX1* LoF variants

**Figure 3:**
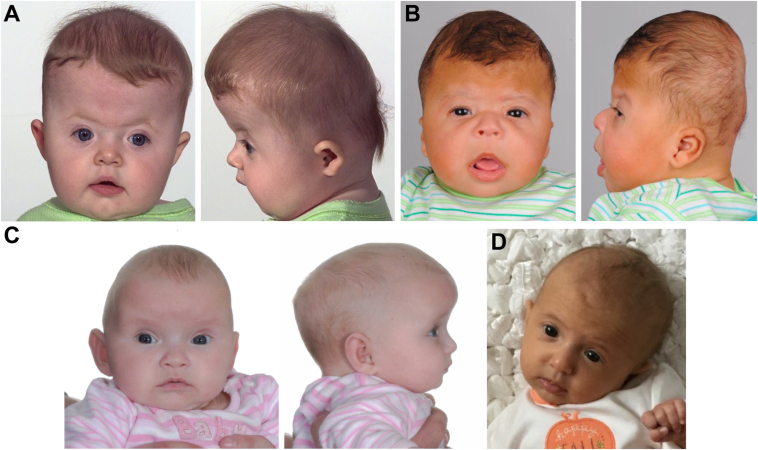
**Preoperative clinical presentation of craniosynostosis in patients with *PRRX1* missense or loss-of-function variants.** A. Individual II-1 from family 2 aged 5 months with nonsyndromic (NS) bicoronal synostosis (p.(Arg18Alafs∗23)). B. Individual III-1 from family 10 aged 3 months with syndromic bicoronal synostosis (p.(Asn144Lys)); this individual also harbors a maternally inherited del(19)(p13.11). C. Individual III-2 from family 5 aged 3 months with NS sagittal synostosis (p.(Arg96Met)). D. Individual III-1 from family 9 aged 2 months with NS right lambdoid synostosis (p.(Arg124Gln)). del, deletion.

We analyzed the craniosynostosis pattern, and associated clinical features, in 17 affected individuals from the 14 families for which we had evidence for pathogenicity (ie, excluding the p.(Asp54Ala) variant). The most frequent presentation (Table 1, Supplemental Table 7) was with synostosis of ≥2 sutures, either pure bicoronal (5 of 17; Figure 3A and B) or other suture combinations (6 of 17). Together this represents an approximately 3-fold excess of multisuture presentations (11 of 17; 65%) compared with approximately 22% for the craniosynostosis population as a whole^4^; single-suture synostoses were sagittal (3 of 17; Figure 3C), unicoronal (2 of 17), and unilambdoid (1 of 17; Figure 3D). Six individuals had required a second major craniofacial procedure (Supplemental Table 8). The associated phenotype is relatively nonspecific, with 10 of 17 individuals classified clinically as nonsyndromic (Supplemental Table 7). Those considered to be syndromic had a variety of additional features (Supplemental Table 8). Family 10, in which the proband and his grandmother had bicoronal synostosis and the grandmother additionally required surgery for unilateral ptosis, had a clinical diagnosis of Saethre-Chotzen syndrome (Figure 3B). One affected individual from family 7, also with bicoronal synostosis, was diagnosed with Pfeiffer syndrome owing to broad thumbs and halluces; in most individuals, the hands and feet were normal. Recurrently noted dysmorphic features included small, posteriorly rotated, or low-set ears (7 subjects) and midface hypoplasia (4 subjects). Cognitive ability was usually in the normal range, except for 2 individuals (III-1 in family 10 and II-1 in family 15) with documented chromosomal abnormalities (respectively, independent of, or including, the *PRRX1* variant). Syndromic diagnoses were otherwise based on minor facial dysmorphic features such as low frontal hairline, eyelid ptosis, prominent orbits, mild midface hypoplasia, and small and/or low-set ears with or without external auditory canal stenosis, which did not amount to a recognizable pattern. Overall, this analysis suggests that the prognosis for affected individuals is good, provided that the consequences of craniosynostosis and any associated intracranial hypertension are addressed, and coincident chromosomal abnormalities are excluded.

To examine the inheritance pattern of the rare *PRRX1* variants, we tested parents and, where available, additional family members. Except for family 2 in which we originally identified the de novo variant, the de novo variant arising in the father of family 9, and the de novo 61.5 kb deletion in family 13, most variants were inherited from parents without craniosynostosis (Table 1, pedigrees shown in Supplemental Figure 1A). Of note, the p.(Asn144Lys) variant (family 10) was inherited from an affected grandmother, but craniosynostosis was not evident in the heterozygous mother; the mother had small ears with external auditory canal stenosis and low anterior hairline. The p.(Arg95∗) variant (family 4) was inherited from a clinically syndromic father, noted to have an atypical skull shape, but who did not present with craniosynostosis. Overall, after excluding the proband in each family, the estimated penetrance of craniosynostosis in other family members proven or deduced to harbor a *PRRX1* variant was 3 of 24 (12.5%); hence, it is apparent that these damaging heterozygous variants are frequently associated with nonpenetrance, and many heterozygous individuals are unaffected.

## Discussion

In this work, we describe the identification of heterozygous LoF and deleterious missense variants of *PRRX1* associated with craniosynostosis in 14 unrelated families. We identified 1 start loss, 3 variants that introduce a premature stop codon (nonsense or frameshift), 6 missense variants (in 7 families) that affect highly conserved residues within the homeodomain, and 3 complete or partial deletions of *PRRX1*. The 6 homeodomain variants reduce nuclear localization of PRRX1 in a cellular assay and were considered pathogenic. In addition, we identified 1 patient with syndromic metopic synostosis and a rare variant outside the homeodomain, p.(Asp54Ala). Notably, Asp54 intersects a putative PRX domain (aa 28-45), which was previously shown to have a subtle inhibitory effect on protein transactivation.^7^ Nevertheless, p.(Asp54Ala) did not abrogate nuclear localization in this study; therefore, the pathogenic significance of this variant remains uncertain. Overall, these observations suggest partial or complete loss-of-function of the variant *PRRX1* allele, consistent with haploinsufficiency as the likely pathogenic mechanism. Interestingly, a small number of individuals have been described with heterozygous deletions of 1q24.2 linked to syndromic learning disability, with short stature, brachydactyly, and facial dysmorphism as additional features.^31^, ^32^, ^33^ Although *PRRX1* falls outside of the shortest region of overlap for the full syndrome, a pair of monozygotic twins has been described with an approximately 2.6 Mb deletion including *PRRX1*, both of whom required surgery for craniosynostosis.^32^

The immunofluorescence analyses suggested a reduced ability of the mutant PRRX1 protein to translocate into the nucleus as a contributing pathogenic mechanism. Four of the 6 missense substitutions reported in this study coincide with 2 predicted nuclear localization sequences within the PRRX1 homeodomain^34^ (Figure 1C) that are located at identical positions to those reported in other members of the homeobox superfamily.^28^^,^^35^^,^^36^ Missense variants at either the N- or C-terminal nuclear localization sequences in PITX2B (also a PRD homeobox transcription factor) also result in abnormal nuclear localization.^36^ In addition, the importance of these 2 regions of the homeodomain is well established for DNA binding. Residues at position 2, 3, and 5-8 on the N-terminal arm and residues 47, 50, 51, 54, and 55 of the homeodomain have all been shown to contribute to DNA-binding specificity^37^; in this study, we identified variants affecting 3 of these critical residues (position 3: p.(Arg96Met), position 51: p.(Asn144Lys), and position 54: p.(Ala147Thr)). Furthermore, in the wider homeobox superfamily literature, variants that correspond in position to the PRRX1 homeodomain missense substitutions have been reported as damaging in multiple disorders (Supplemental Table 9). Surprisingly, we also found abnormal nuclear localization of the p.(Arg115Trp) and p.(Arg124Gln) variants. Substitutions at these positions are predicted to affect DNA binding by PredictProtein software,^34^ and the equivalent position to Arg124 in the homeobox protein PROP1 was reported to disrupt this function (Supplemental Table 9). Both residues reside within the major alpha helices in PRRX1 (Figure 1C), with residue 31 of the homeodomain (Arg124) interacting with Glu135 in the recognition helix and capable of forming homeodomain-DNA salt bridges.^38^ Changes at residue 22 (Arg115) have been hypothesized to affect protein interaction with co-activators,^39^ possibly reducing efficiency of nuclear import. Although we did not assay DNA-binding activity, an impaired ability to enter the nucleus would be sufficient to abrogate PRRX1-mediated gene regulation. In this study, we prioritized analysis of variants identified in patients with craniosynostosis; however, there are several other rare homeodomain missense variants reported in population databases, such as gnomAD and UK Biobank, that could be disease causing. Future clinical and functional studies could explore further the effect of rare missense variation across the homeodomain to establish any hidden genotype-phenotype correlation. Additionally, though variants outside of the homeodomain could disrupt protein transactivation (as hypothesized for the p.(Asp54Ala) variant), the 3.5-fold enrichment of rare variants within the homeodomain (based on our resequencing survey), combined with the low disease penetrance of pathogenic variants, indicates that establishing disease causality for variants outside this well-studied domain would be challenging.

The phenotype associated with *PRRX1* pathogenic variants and craniosynostosis seems relatively nonspecific, with a variety of sutures fused and no diagnostic syndromic features. However, there was a clear tendency to fusion of multiple sutures—especially bicoronal and other multisuture presentations, which in combination were 3-fold over-represented compared with their occurrence in craniosynostosis as a whole. The requirement for repeated craniofacial procedures (6 of 17 individuals) was also high. Together, these considerations indicate that a diagnosis of *PRRX1*-related craniosynostosis serves as a prognostic marker of greater severity and suggests that children harboring pathogenic *PRRX1* variants should be monitored throughout their growth for possible late development of raised intracranial pressure. Based on the aggregated data from the 3 craniosynostosis cohorts that we screened, the prevalence of pathogenic *PRRX1* variants was 0.76% (11 of 1455; Supplemental Table 1). However, this figure is subject to potential bias, for example, the mix of subjects screened was not based on a cross-sectional population sample, and only a subset of the total (*n* = 479) was screened for deletions. A lower prevalence of 0.3% (3 of 981) was determined from a prospective UK 18-year birth cohort of 981 affected individuals (unpublished); however, the 95% CI is wide (0.06%-0.9%). Hence, we anticipate that the screening of other large population cohorts should reveal further affected individuals with *PRRX1* variants, alongside unaffected family members. An important consideration for variant interpretation is that the penetrance of pathogenic variants in the heterozygous state is low (12.5%; 3 of 24 relatives of the proband who are heterozygous for the pathogenic variant are affected, Supplemental Figure 1). The LoF intolerance score (pLI) and LoF observed/expected upper bound fraction are 0.24 and 0.66, respectively.^26^ These moderate constraint values are concordant with the deduction that haploinsufficiency can cause significant congenital disease but with reduced penetrance. Hence, transmission of a *PRRX1* variant from an unaffected parent does not exclude a causal connection in an individual with craniosynostosis. The variable phenotype is likely attributable to a complex combination of genetic factors (as discussed below) and environmental differences, including fetal head constraint.^40^

An important and currently unresolved question is how our observations can be reconciled with previous reports of 3 heterozygous *PRRX1* variants^16^^,^^18^^,^^19^ and, less problematically, 1 homozygous variant^17^ associated with the much more severe phenotype of agnathia-otocephaly. The homozygous variant (NM_022716.4:c.691G>C; p.(Ala231Pro)) could be consistent with substantially reduced function of PRRX1 leading, as in the mouse,^15^ to severe deficiency of facial structures. The association of the heterozygous variants with agnathia-otocephaly is harder to explain in the light of our new data, particularly given that 2 of the 3 variants predict frameshifts (NM_022716.4:c.266_269dup; p.(Arg92Glufs∗8),^18^ and NM_022716.4:c.269del; p.(Lys90Argfs∗42))^19^ in the protein occurring very close to nonsense and frameshift variants (p.(Arg95∗) and p.(Asn97Lysfs∗35)) reported here (Figure 1B). Importantly, none of the 3 individuals with partial or complete deletions of *PRRX1* (families 13-15) had features of agnathia-otocephaly, excluding haploinsufficiency as the mechanism of this more severe phenotype. Moreover, we found no convincing evidence that the p.(Phe113Leu) missense variant (identified in a patient with agnathia-otocephaly) affected nuclear import (Supplemental Figure 5), although this does not exclude other pathogenic mechanisms. Differences in genetic background might contribute to the discrepant phenotypes, which could be either allelic differences (for example, relative expressivity of mutant and wild-type *PRRX1* allele, as observed in the case of *RBM8A* pathogenic variants in thrombocytopenia-absent radius syndrome)^41^ or nonallelic differences; although beyond the scope of this work, it would be interesting to explore possible allelic modifiers in further studies. Additionally, given prior evidence from the mouse of functional redundancy between *Prrx1* and *Prrx2*,^15^ coincident LoF pathogenic variants in *PRRX2* (which is not currently recognized as a Mendelian disease gene) could have exacerbated the phenotype. Further comments on these potential explanations are provided in Supplemental Table 10. We contacted the authors of all the previous reports with the aim of undertaking comparative genomic studies. Unfortunately, a lack of suitable biological materials from the individuals with agnathia-otocephaly, related to early lethality, has hampered a more systematic approach to resolving this problem.

Based both on our own functional analyses and the observation of phenotypes associated with 1q24.2 deletions (discussed above), we conclude that heterozygous LoF and missense pathogenic variants within the homeodomain of *PRRX1* are normally associated with craniosynostosis or milder/normal phenotypes. Independent analyses have shown that *Prrx1* is expressed in the major cranial sutures of the mouse, within the intrasutural mesenchyme but absent from the osteogenic fronts.^8^^,^^9^^,^^11^^,^^42^ Fate mapping showed that the cells expressing *Prrx1* later differentiated into osteoblasts lining the bony plate surfaces. It was concluded that *Prrx1* expression characterizes a subpopulation of cells spanning the late stem cell (Sca1+) and early osteogenic (Runx2+) stages.^9^ PRRX1 may link to the BMP-MSX2 signaling axis in the cranial suture, overactivity of which has previously been implicated in craniosynostosis.^43^^,^^44^ Binding of PRRX1 to a specific sequence within the promoter of *Msx2* was demonstrated in vitro,^45^ and in the mouse mandible, it has been shown that the requirement for a high level of BMP4 to induce expression of the homeobox gene *Msx2* can be bypassed in a Prrx1^−/−^;Prrx2^−/−^ genetic background, indicating that PRRX1 acts as a repressor in this context^46^; hence, if the same activity occurs in the cranial suture, the effect of a *PRRX1* pathogenic variant would be to alleviate this repression, thus predisposing to craniosynostosis. In summary, to our knowledge, our new human genetic findings complement these mouse studies, confirming the important role of PRRX1 in cranial suture function by providing the first genetic evidence that approximately 50% reduction in PRRX1 levels is sufficient, in some individuals, to lead to craniosynostosis.

## Data Availability

All data are available in the manuscript and supplemental material.

## Conflict of Interest

The authors declare no conflicts of interest.
