# Supplementary material for: Pathogenic variants in the paired-related homeobox 1 gene (PRRX1) cause craniosynostosis with incomplete penetrance
Source: Genet Med. 2023 Sep;25(9):100883. doi: 10.1016/j.gim.2023.100883 (PMC11554955; doi:10.1016/j.gim.2023.100883)
Supplement: Supplementary Information [file mmc2.pdf]

Pathogenic variants in the paired-related homeobox 1 gene (*PRRX1*) cause craniosynostosis with incomplete penetrance

Rebecca S. Tooze, Kerry A. Miller, Sigrid M. A. Swagemakers, *et al*.

Supplemental Methods

Screening individuals with craniosynostosis

Primers were designed to screen all coding exons of *PRRX1* (Supplemental Table 2) in 388 samples. A 20 ng sample of DNA was mixed with each primer (at a final concentration of 0.5 µM per primer), 1x Q5 buffer, 200 µM each dNTP and 0.5 U of Q5® high-fidelity DNA polymerase (New England BioLabs). Cycling conditions consisted of a 30 s denaturation step at 98°C, followed by 35 cycles of 98°C for 10 s, 60–65°C for 30 s (details in Supplemental Table 2), and 72°C for 30 s, followed by a final extension step of 72°C for 10 min. Illumina-specific sequence adaptors and 10 bp sample indexes were attached using the Access Array™ Barcode Library for Illumina® Sequencers-384, Single Direction (Fluidigm), at a concentration of 0.4 µM per primer with Q5® high-fidelity DNA polymerase as above, for 9 cycles only. Indexed PCR products were pooled, purified with AxyPrep MAG PCR Clean-Up Kit (Axygen) and quantified using the 2200 TapeStation (Agilent Technologies) with a High Sensitivity D1000 ScreenTape and a Qubit® 1.0 Fluorometer (ThermoFisher Scientific), following the manufacturer's instructions. Pooled and indexed PCR products were diluted to a final concentration of 9 pM and sequenced using the Illumina MiSeq platform with a MiSeq Reagent Kit v2 for 500 cycles (Illumina) according to the manufacturer's instructions, before analysis using amplimap software (including mapping, coverage analysis and variant calls).[1]

Targeted resequencing

Analysis of 541 samples with undiagnosed craniosynostosis (considering a variety of sutures fused) for variants in *PRRX1* was undertaken using IDT's hybridization and capture protocol (version 4, May 2019). Individual libraries for capture-based resequencing were prepared using the 'Twist Bioscience' kits for the enzymatic fragmentation and universal adapter system (protocol version 11 (September 2019) Rev1) or the 'xGenTM DNA Library Prep EZ Kit' and protocol (IDT, protocol version 1 (December 2021)). DNA was fragmented to ~200 bp,

before adding adapters and indexing primers, and subsequently analysed using broad-range Qubit and D1000 TapeStation reagents (average fragment size of 330 bp). The prepared libraries were pooled to a total of 6 μg of DNA and between 32–40 samples per hybridization capture reaction. The hybridization reactions were carried out at 65°C for 16 hours. After hybridization, the pooled libraries were washed and post-capture PCR was performed following manufacturer's protocol (IDT xGen hybridization capture of DNA libraries for NGS target enrichment, 7 PCR cycles were used), for a panel containing 2054 probes. The amplified capture reactions were washed with beads before quantification and validation using high-sensitivity (HS) qubit reagents and HS D1000 TapeStation, and next generation sequencing analysis using Miseq v3 (150-cycle, MRC WIMM Sequencing Facility). Data were analyzed using amplimap software.[1]

Coverage analysis

In a subset of patients (n = 479) included in the targeted resequencing analysis, coverage was assessed. Normalized coverage was calculated by dividing the coverage for each gene by the mean coverage of all genes (n = 41) screened in a given sample. This value was normalized to the average coverage for all samples for a given gene. The normalized mean coverage for the gene with the largest deviation from 1 was removed for each sample before the mean and standard deviation (SD) of coverage for all other genes was calculated. After ranking each sample by SD, the error behaviour was calculated as SD_b-SD_a, where a and b correspond to ranked sample numbers. Data quality noticeably deteriorated at n = 479, which corresponded to a SD threshold of 0.045, and all samples with a SD value above 0.045 were removed from the analysis. The two deletions identified were independently confirmed using array comparative genomic hybridization; individual II-2 in Family 14 was reported to harbor the rearrangement arr 1q24.2(170,622,833-170,688,644)x1 [hg19] and II-1 in Family 15 had arr 1q24.2q25.2(167,699,849-178,212,109)x1 [hg19].

Site-directed mutagenesis

A vector containing *Prrx1a* was obtained from Michael Kern's laboratory. Site-directed mutagenesis was conducted using two methods. For the R124Q and N144K plasmids, overlapping primers were designed containing the variant nucleotide in the centre of both complementary primers. Initially, 40 ng of plasmid DNA was added to 1.5 μl of each 10 μM

primer (Supplemental Table 5), 1 µl of 10 mM dNTPs, 10 µl of Q5 buffer and enhancer, 1 µl of Q5 polymerase and up to 50 µl of nuclease-free water. Reactions were placed in the Thermal Cycler (BIORAD T100TM) at 95°C for 2 min, followed by 95°C for 30 s, 55°C for 1 min and 72°C for 10 min, for 12 cycles. Amplicons were digested with DpnI (NEB, #R0176) in CutSmart Buffer for 4 hours at 37°C. Digested products were transformed into competent *E. coli* (NEB 5-alpha, C2987) using the manufacturers 'High Efficiency Transformation Protocol'. Plasmid DNA was extracted using a miniprep kit (QIAprep Spin 27106, QIAGEN) and sent for dideoxy-sequencing. For all remaining vectors, site-directed mutagenesis was conducted following the NEB Q5 site-directed mutagenesis kit quick protocol (E0554). For these vectors, 25 ng of template DNA was added to 12.5 µl Q5 hot start high-fidelity 2x master mix, 0.5 µM of each forward and reverse primer (Supplemental Table 5), and up to 25 µl of nuclease-free water. The reactions were placed in a thermocycler under the following conditions: 98°C for 30 s, followed by 25 cycles of 98°C for 10 s, 58–72°C (as documented in Supplemental Table 5) for 20 s and 72°C for 5 min; a final extension of 72°C for 2 min was performed. Following site directed mutagenesis, 1 µl of product was incubated with 5 µl of kinase, ligase, and DpnI (KLD) 2x reaction buffer (NEB, E0554S), 1 µl of 10x KLD enzyme mix, and 3 µl of nuclease-free water for 5 min at room temperature. Finally, 5 µl of the KLD reaction was transformed into 50 µl of chemically competent cells (NEB 5-alpha, C2987H) following manufacturer's guidelines. The presence of all variants was confirmed by dideoxy-sequencing (Supplemental Figure 3).

Sub-cloning into a HA-tagged vector

Prrx1 cDNA was amplified using a Q5 polymerase protocol (1 µg of plasmid DNA [pcDNA3.1-*Prrx1a*], 12.5 µl Q5 hot start 2x master mix, 10 µM of each forward [Prrx1a_mouse_EcoRI: 5'-GAATTCATGACCTCCAGCTACGGGCACGTTC-3'] and reverse primer [Prrx1a_mouse_HindIII: 5'-AAGCTTCAGTTGACTGTTGGCACCTGGTTCCTC-3'], to a final volume of 25 µl with nuclease-free water). Products were amplified in a thermocycler at 98°C for 30 s, followed by 35 cycles of 98°C for 10 s, 70°C for 20 s and 72°C for 30 s, with a final extension of 72°C for 2 min. A HA and FLAG tagged plasmid was obtained from Addgene (FLAG-HA-pcDNA3.1, #52535) and digested, alongside the amplified PCR products (1 µg of plasmid or PCR product), using 1 µl of each HindIII-HF and EcoRI-HF in 1x CutSmart Buffer. The products were incubated with enzyme at 37°C for 15 min before adding 1 µl of alkaline phosphatase for a further 15 min.

Digested products were run on a 1% agarose gel and gel purified (NEB, T1020 gel extraction kit). Purified *Prrx1a* inserts were ligated into the cut FLAG-HA-pcDNA3.1 plasmid using a quick ligation protocol (NEB, M2200) and a 4:1 ratio of insert DNA to vector (μl). Ligated products were transformed into one shot *Stbl3* chemically competent cells (Invitrogen, C7373-03) following the manufacturer's protocol. Colonies were screened by colony PCR and positive colonies were cultured in LB, supplemented with ampicillin (50 μg/ml). The plasmid was purified using a maxiprep kit (Qiagen, 12162) before sequence confirmation by dideoxy sequencing.

The S104G and R115W variants were introduced directly into the HA/FLAG-tagged vector using the Q5 site-directed mutagenesis kit (NEB, E0554S) as described previously for site-directed mutagenesis of the initial *Prrx1a* vector inherited from the Kern lab. An annealing temperature of 65°C or 70°C was used for S104G and R115W, respectively.

Supplemental Table 1 Phenotypic composition of cohorts of unsolved CRS probands

	Complete Genomics Trios[b]		Clinical Genetics Group (Oxford) Resequencing Cohort		Yale WES Cohort		Totals		
**Phenotype[a]**	*PRRX1*-negative	*PRRX1*-positive	*PRRX1*-negative	*PRRX1*-Positive	*PRRX1*-negative	*PRRX1*-positive	***PRRX1*-negative**	***PRRX1*-positive**	**Percent (%) positive**
**NS metopic**			152		169		321		
**NS unicoronal**			178	2			178	2	1.11
**NS sagittal**			312	1	298		610	1	0.16
**NS unilambdoid**			10		18	1	28	1	3.45
**NS multiple**			51	3	16		67	3	4.29
**NS bicoronal**	1	1	31				32	1	3.03
**NS bilambdoid**			2		2		4		
**S metopic**			40	1	5		45	1	2.17
**S unicoronal**			24				24		
**S sagittal**	1		50		6		57		
**S unilambdoid**			2		1		3		
**S multiple**	1	1	40		4		45	1	2.17
**S bicoronal**	1		9	1			10	1	9.09
**S bilambdoid**			1				1		
**Carpenter**			1				1		
**Crouzon**			4				4		
**Pfeiffer**			3				3		
**Saethre Chotzen**			10				10		
**Other**			1				1		
**Total**	**4**	**2**	**921**	**8**	**519**	**1**	**1444**	**11**	**0.76%**

[a]NS = non-syndromic, S = syndromic

[b]9 cases were originally sequenced but 3 have since been solved and removed from the total numbers in this table

Supplemental Table 2 Primers used (excluding CS tags) for sequencing of gDNA

ID	Target Length (bp)	Forward Primer (5' – 3')	Reverse Primer (5' – 3')	Tm (°C)
PRRX1-Ex1a	406	ATCTCTTTGGACCGCGCC	CTAGCAGGTGACTGACGGAG	65
PRRX1-Ex1b	378	AGACCATGACCTCCAGCTAC	TGAAGTCTGCCATCACCTCTC	62
PRRX1-Ex2	390	GATGTGAGCAAATGAAGCAAG	GCATGAGCCACTGTGCTG	65
PRRX1-Ex3	397	ACGGAGAATTCCATAGCCATC	AGCAGCTTGAAACATGACCG	60
PRRX1-Ex4	388	AGTGAATGGCCTGGTTTTGC	TTCTAGAACTGCAACCCCCAC	60
PRRX1-Ex5	453	TCCCTTTCCTCACTCTACACC	GCAGATGAAGAAATAACAGAGCAG	60

Supplemental Table 3 Primers used in a targeted enrichment analysis

Chr	Start Position (GRCh38)	End Position (GRCh38)	ID
chr1	170664188	170664308	Target029_PRRX1_ENST00000239461.11_2
chr1	170664248	170664368	Target029_PRRX1_ENST00000239461.11_3
chr1	170664308	170664428	Target029_PRRX1_ENST00000239461.11_4
chr1	170664368	170664488	Target029_PRRX1_ENST00000239461.11_5
chr1	170664428	170664548	Target029_PRRX1_ENST00000239461.11_6
chr1	170719663	170719783	Target030_PRRX1_ENST00000239461.11_1
chr1	170719723	170719843	Target030_PRRX1_ENST00000239461.11_2
chr1	170719783	170719903	Target030_PRRX1_ENST00000239461.11_3
chr1	170719843	170719963	Target030_PRRX1_ENST00000239461.11_4
chr1	170726130	170726250	Target031_PRRX1_ENST00000239461.11_1
chr1	170726190	170726310	Target031_PRRX1_ENST00000239461.11_2
chr1	170726250	170726370	Target031_PRRX1_ENST00000239461.11_3
chr1	170726310	170726430	Target031_PRRX1_ENST00000239461.11_4
chr1	170726370	170726490	Target031_PRRX1_ENST00000239461.11_5
chr1	170730214	170730334	Target031b_PRRX1_ENST00000367760.7_1
chr1	170730274	170730394	Target031b_PRRX1_ENST00000367760.7_2
chr1	170735967	170736087	Target032_PRRX1_ENST00000239461.11_1
chr1	170736027	170736147	Target032_PRRX1_ENST00000239461.11_2
chr1	170736087	170736207	Target032_PRRX1_ENST00000239461.11_3
chr1	170736147	170736267	Target032_PRRX1_ENST00000239461.11_4

Supplemental Table 4 Primers used for isolating breakpoints of *PRRX1* deletions

ID	Forward Primer (5' – 3')	Reverse Primer (5' – 3')	Tm (°C)
Exons 1–2[a]	GCAGTGTATCGGAGTTTCAGTTGCTTG	CCAGCTATCTCTGTGGTAGCTCAATC	66
Exons 2–5[b]	CAGGATCAGGAAGCCCTTTCTAATAACTGAC	CTTCCTCAGTTTGTATTTGGTATTGACATT	66

[a]For analysis of Family 14 (76 kb deletion)
[b]For analysis of Family 13 (61.5 kb deletion)

Supplemental Table 5 Mutagenesis primers

ID	Forward Primer (5' – 3')[a]	Reverse Primer (5' – 3')[a,b]	Tm (°C)
PRRX1-D54A	GCACAAGCAG**C**CGAAAGTGTGGGCG	CGCCACCATGTCCCCGGC	72
PRRX1-R96M	AAGCAGCGGA**TG**AACAGGACAAC	TCTCTTCTTCTTCTCCTC	57
PRRX1-S104G	ATTCAACAGC**G**GCCAACTGCA	GTTGTCCTGTTTCTCCGC	65
PRRX1-F113L	GGAGCGTGTC**C**TTGAGCGGAC	AAGGCCTGCAGTTGGCTG	70
PRRX1-R115W	TGTCTTTGAG**T**GGACACATTACCCG	CGCTCCAAGGCCTGCAGT	70
PRRX1-R124Q	CCCGGATGCTTTTGTTC**A**AGAA<br>GATCTCGCACGTC	GACGTGCGAGATCTTCT**T**GAA<br>CAAAAGCATCCGGG	66
PRRX1-N144K	GCAGGTGTGGTTTCAGAA**G**CGA<br>AGAGCCAAGTTC	GAACTTGGCTCTTCG**C**TTCTGA<br>AACCACACCTGC	66
PRRX1-A147T	GAACCGAAGA**A**CCAAGTTCCG	TGAAACCACACCTGCACT	65
PRRX1-R150H	GCCAAGTTCC**A**CAGGAATGAG	TCTTCGGTTCTGAAACCAC	63

[a]Variant substitutions are highlighted in bold and underlined
[b]Site-directed mutagenesis of R124Q and N144K were completed using complementary overlapping primer pairs while the remaining variants were constructed using a Q5 site-directed mutagenesis kit (NEB, E0554) with abutting primers pairs (only the forward primer contains the variant substitution).

Supplemental Table 6 Primers used for confirming plasmid sequence

ID	Primer (5' – 3')	Tm (°C)
PRRX1.cDNA.2F	GGCAACCTCGACACCCTG	65
PRRX1.cDNA.6R	CTGTGCAGGGCTATTGTTGG	58
CMV_FW	CGCAAATGGGCGGTAGGCGTG	65
T7_FW	TAATACGACTCACTATAGGG	50
BGH_RV	TAGAAGGCACAGTCGAGG	55
*Prrx1a*_mouse_EcoRI	GAATTCATGACCTCCAGCTACGGGCACGTTC	67
*Prrx1a*_mouse_HindIII	AAGCTTCAGTTGACTGTTGGCACCTGGTTCCTC	68

Supplemental Table 7 Patterns of suture fusion and presence of syndromic features in individuals heterozygous for functionally significant *PRRX1* variants

		**Start-lost**	**Arg18Alafs*23**	**Arg95***	**Arg96Met**	**Arg96Met**	**Asn97Lysfs*35 III-1**	**Asn97Lysfs*35 III-2**	**Arg115Trp III-1**	**Arg115Trp III-3**	**Arg124Gln**	**Asn144Lys I-2**	**Asn144Lys III-1**	**Ala147Thr**	**Arg150His**	**61.5 kb deletion**	**76 kb deletion**	**10.5 Mb deletion**	**Total**
	**Family #**	**1**	**2**	**4**	**5**	**6**	**7**	**7**	**8**	**8**	**9**	**10**	**10**	**11**	**12**	**13**	**14**	**15**	
Suture(s) fused[a]	Metopic																		**0**
	Left Coronal		**X**				**X**					**X**	**X**	**X**	**X**		**X**	**X**	**8**
	Right Coronal		**X**	**X**		**X**	**X**					**X**	**X**	**X**	**X**			**X**	**9**
	Sagittal	**X**		**X**	**X**				**X**	**X**				**X**	**X**				**7**
	Left Lambdoid	**X**																	**1**
	Right Lambdoid										**X**								**1**
	Multi-suture							**X**								**X**			**2**
	Syndromic?						**Y**		**Y**	**Y**		**Y**	**Y**		**Y**			**Y**	**7**

[a]Y = yes, X = presence of suture fusion

Supplemental Table 9 Variants described within the homeodomain of homeobox transcription factors at positions corresponding to those in PRRX1.

Gene (UniProt ID)	Position in homeodomain	Genomic Coordinates (GRCh38)	cDNA	aa Change	Classification[a]	Identical aa change to this study?[b]	Functional Data/ Additional Information
CRX (O43186)	3	chr19: 47836263<br><br>chr19: 47836264	NM_000554.6: c.121C>T<br><br>NM_000554.6:c.122G>A	p.(Arg41Trp)<br><br>p.(Arg41Gln)	Likely pathogenic (PS3 – Strong, PM1 – Moderate, PP1 - Supporting)<br><br>Likely pathogenic (PS3 – Strong, PM1 – Moderate, PM5 – Moderate, PP4 – Supporting)	No	Decreased DNA binding activity in mutant compared to wild-type (WT) as illustrated by an electrophoretic mobility shift assay (EMSA).[2]
HESX1 (Q9UBX0)	3	chr3: 57198784	NM_003865.3:c.326G>A	p.(Arg109Gln)	Likely pathogenic (PS3 – Strong, PM1 – Moderate, PM6 - Moderate)	No	Nuclear localization, as illustrated through immunofluorescence, showed no difference between WT and mutant *HESX1.* DNA-binding ability was abrogated in the mutant using an EMSA.[3]
PHOX2B (Q99453)	3	chr4: 41747479	NM_003924.4:c.299G>T	p.(Arg100Leu)	Likely pathogenic (PM1 – Moderate, PM2 – Moderate, PP1 – Supporting, PP3 - Supporting)	No	No functional analysis. Heterozygous variant predicted damaging owing to position in homeodomain.[4]
SHOX (O15266)	3	chrX: 634695	NM_000451.4:c.355C>G	p.(Arg119Gly)	Likely pathogenic (PM1 – Moderate, PM2 – Moderate, PP1 – Supporting, PP4 - Supporting)	No	No functional analysis. Missense variant predicted damaging owing to change in charge.[5]
POU4F1 (Q01851)	22	-	-	Experimental substitution to isoleucine[c]	-	No	This variant is not described in a patient. The authors substituted the 20th position of the

							homeodomain to isoleucine in *POU4F1*, which resulted in a decrease in promoter activity.[6]
MSX2 (P35548)	31	chr5: 174729294	NM_002449.5:c.515G>A	p.(Arg172His)	Pathogenic (PS1 – Strong, PS3 – Strong, PM1 – Moderate, PP1 – Supporting)	No	Mutant bound target sequence with an affinity 15% that of WT.[7]
PROP1 (O75360)	31	chr5: 177994152	NM_006261.5:c.296G>A	p.(Arg99Gln)	Likely pathogenic (PS3 – Strong, PM1 – Moderate, PP1 – Supporting)	Yes p.(Arg124Gln)	Reduced DNA binding activity in mutant compared to WT as demonstrated via an EMSA. Mutant was unable to activate the reporter in a luciferase assay.[8]
SHOX (O15266)	31	chrX: 634780<br><br>chrX: 634780	NM_000451.4:c.440G>A<br><br>NM_000451.4:c.440G>C	p.(Arg147His)<br><br>p.(Arg147Pro)	Likely pathogenic (PM1 – Moderate, PM2 – Moderate, PM5 – Moderate, PM6 – Moderate, PP4 - Supporting)<br><br>Likely pathogenic (PM1 – Moderate, PM2 – Moderate, PM6 – Moderate, PP3 – Supporting, PP4 - Supporting)	No	No functional analysis but considered damaging owing to conservation and position in the homeodomain.[9]<br><br>No functional analysis; heterozygous variant considered damaging owing to conservation and PolyPhen score.[10]
HOXA13 (P31271)	51	chr7:27198251	NM_000522.5:c.1114A>C	p.(Asn372His)	Likely pathogenic (PS2 – Strong, PM1 – Moderate, PM2 – Moderate)	No	No functional analysis but considered damaging owing to position within the homeodomain and presence of a *de novo* variant in the affected individual.[11]

LMX1B (O60663)	51	chr9:129455868	NM_001174146:c.807C>A	p.(Asn269Lys)	Likely pathogenic (PS2 – Strong, PM1 – Moderate, PM2 – Moderate)	Yes p.(Asn144Lys)	No DNA binding activity in mutant yet strong activity in WT.[12]
SHOX (O15266)	51	chrX:640833	NM_000451.4:c.499A>G	p.(Asn167Asp)	Likely pathogenic (PM1 – Moderate, PM2 – Moderate, PM6 – Moderate, PP3 – Supporting)	No	No functional analysis but considered damaging by Alamut software.[13]
LHX3 (Q9UBR4)	54	chr9: 136198798	NM_178138.6:c.629C>T	p.(Ala210Val)	Likely pathogenic (PS3 – Strong, PM1 – Moderate, PM2 – Moderate, PP4 - Supporting)	No	Homozygous variant. Significantly reduced DNA binding activity in mutant compared to WT as shown by an EMSA.[14]
PROP1 (O75360)	54	chr5:177993017	NM_006261.5:c.373C>T	p.(Ala125Trp)	Likely pathogenic (PM1 – Moderate, PM2 – Moderate, PM3 – Moderate, PP1 – Supporting)	No	WT and mutant plasmids were indifferent when nuclear localization was assessed. The mutant retained ability to bind DNA, as shown via an EMSA, but showed faster migration. There was a subtle reduction in luciferase reporter activity in the mutant compared to WT. This patient is compound heterozygous for this missense variant and an indel resulting in a frameshift (c.310delC).[15]
SHOX (O15266)	54	chrX: 640842<br><br>chrX: 640843	NM_000451.4:c.508G>C<br><br>NM_000451.4:c.509C>A	p.(Ala170Pro)<br><br>p.(Ala170Asp)	Likely pathogenic (PS3 – Strong, PM1 – Moderate, PM2 – Moderate, PP4 – Supporting)<br><br>Likely pathogenic (PM1 – Moderate, PM2 – Moderate, PP1 –	No	Both homozygous and heterozygous variants reported. *SHOX* mutants show abnormal nuclear localization.[16,17]

					Supporting, PP4 – Supporting)		
RAX (Q9Y2V3)	57	chr18: 59269470	NM_013435.3:c.575G>A	p.(Arg192Gln)	Pathogenic (PS3 – Strong, PM1 – Moderate, PM2 – Moderate, PM3 – Moderate)	No	Compound heterozygous for the missense variant and p.(Gln147*). The missense variant showed reduced DNA binding activity compared to the WT, as illustrated by an EMSA.[18]
SHOX (O15266)	57	chrX: 640851	NM_000451.4:c.517C>T	p.(Arg173Cys)	Pathogenic (PS3 – Strong, PM1 – Moderate, PM2 – Moderate, PM5 – Moderate, PM6 – Moderate, PP4 – Supporting)	Yes p.(Arg150His)	*SHOX* mutants show abnormal nuclear localization and no DNA binding activity in an EMSA.[19]
		chrX: 640852	NM_000451.4:c.518G>A	p.(Arg173His)	Likely pathogenic (PS3 – Strong, PM1 – Moderate, PM5 – Moderate, PM6 – Moderate, PP4 – Supporting)		*SHOX* mutants show abnormal nuclear localization and no DNA binding activity in an EMSA.[19]
		chrX: 640852	NM_000451.4:c.518G>T	p.(Arg173Leu)	Likely pathogenic (PM1 – Moderate, PM2 – Moderate, PM5 – Moderate, PM6 – Moderate, PP4 – Supporting)		No functional analysis but considered damaging owing to its position within the homeodomain.[13]

[a]Variants are classified based on ACMG criteria: https://www.acgs.uk.com/media/11631/uk-practice-guidelines-for-variant-classification-v4-01-2020.pdf
[b]See table 1 for additional information on the variants described in *PRRX1* in this study.
[c]In *PRRX1* residue 22 encodes arginine.

Supplemental Table 10 Potential explanations for the more severe agnathia-otocephaly phenotypes associated with heterozygous *PRRX1* variants.

Explanation	Comments
A variant present on the *trans*- allele was not identified	All 3 reports state that the entire coding region of *PRRX1* was sequenced; however, non-coding variants or a partial deletion of the *trans* allele (separated from the site of the heterozygous substitution) could have been missed
The normal *trans*- allele harboured sequences (in the 5' or 3' UTR or in intronic or flanking regions in *cis*) causing reduced expression of this allele	Significant (P = 4 x 10^{-9}) cis-eQTL rs56250774 with effect size -0.57 identified for *PRRX1* in anterior cingulate cortex (GTex consortium)
An additional loss of function variant was present in the *PRRX2* gene	1. In support of this, synergism between *Prrx1* and *Prrx2* is particularly evident for the mandible, which is severely reduced in *Prrx1*$^{-/-}$;*Prrx2*$^{-/-}$ double knockout mice[20,21]<br>2. The constraint score of *PRRX2* (pLI) = 0.31 (1 LoF observed, 3.8 expected); no common (>10^{-4}) LoF alleles identified in gnomAD
Variation in expression of *PRRX2* influences the phenotype	Significant (P = 8 x 10^{-8}) cis-eQTL rs11788582 with effect size -0.21 identified for *PRRX2* in tibial artery (GTex consortium)
Other factors in the genetic background	Variants in *OTX2* are more frequent than *PRRX1* as a cause of agnathia-otocephaly[22,23]

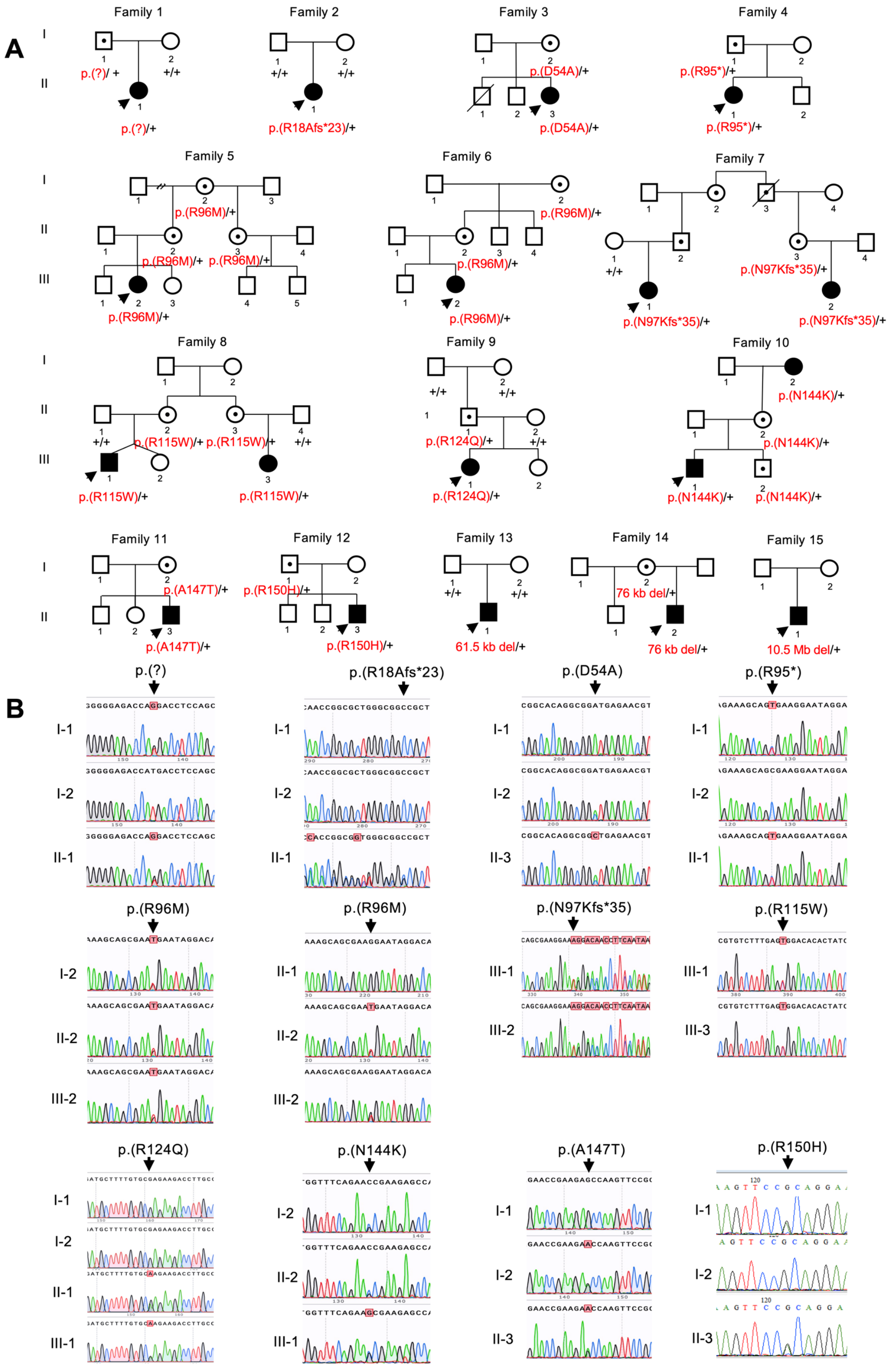

Supplemental Figure 1 Pedigree figures and dideoxy sequencing confirmation of each variant. (A) Pedigrees of all families in which a small nucleotide variant or deletion was identified in *PRRX1*. The index patient is denoted by the arrowhead. If available, inheritance of the variant from family members have been confirmed by sequencing and documented on the pedigree (red lettering). A dot in the middle of the pedigree symbol indicates a heterozygote family member. (B) Confirmation of small nucleotide variants (by dideoxy-sequencing) identified by whole genome sequencing and targeted resequencing analyses (PCR and hybridization capture). Sanger traces show a representative result from forward or reverse sequencing. Arrows indicate the position of each variant.

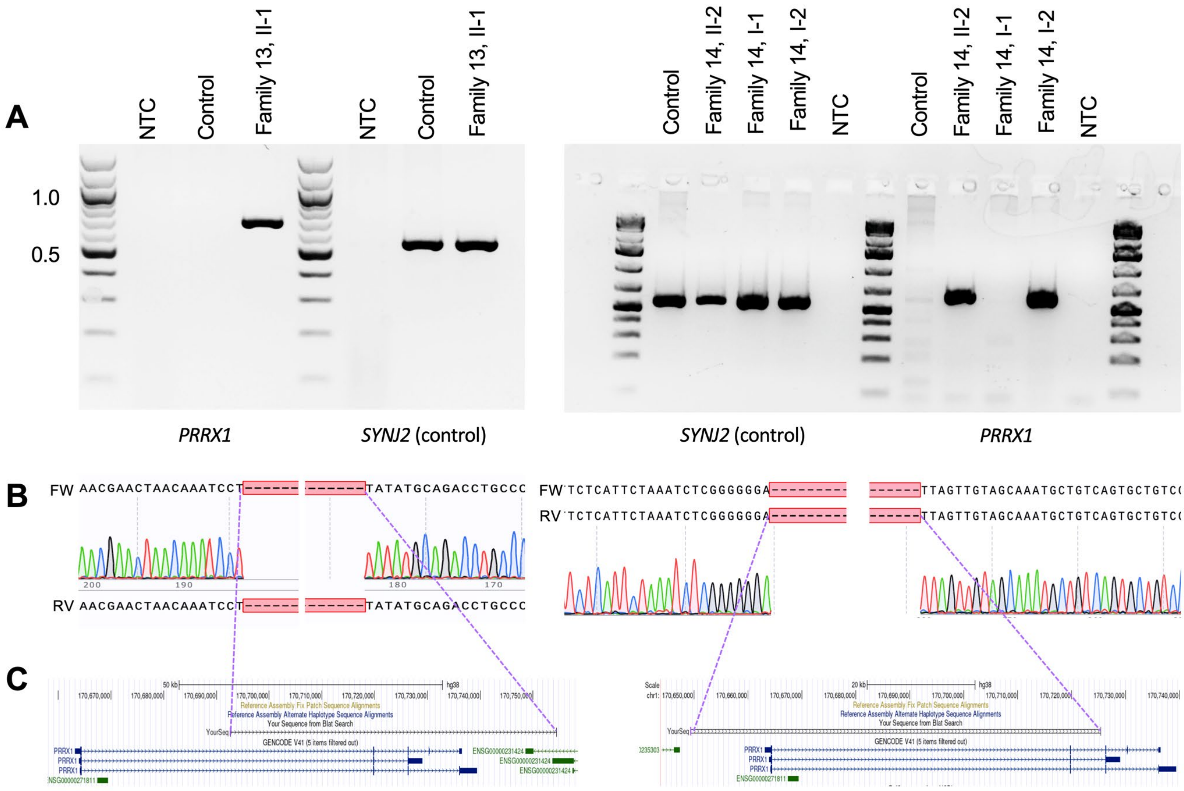

Supplemental Figure 2 Analysis of the breakpoints of partial deletions including *PRRX1*. (A, B) Left, breakpoints in Family 13; right, breakpoints in Family 14. (A) Breakpoint PCR of the affected proband, available family members, and a healthy control showing presence of the amplified product using *PRRX1* primers in both families with the deletion, but no product in the healthy control. Primers amplifying a region of *SYNJ2* (552 bp) were used to confirm that the control sample could be amplified. NTC = no template control. (B) Forward (FW) and reverse (RV) dideoxy sequencing traces showing the region of the respective 3' and 5' breakpoints. (C) A screenshot from UCSC genome browser indicating the region of *PRRX1* where the two breakpoints align (GRCh38: left = chr1:170692716_170754397del, right = chr1:170649419_170725333del).

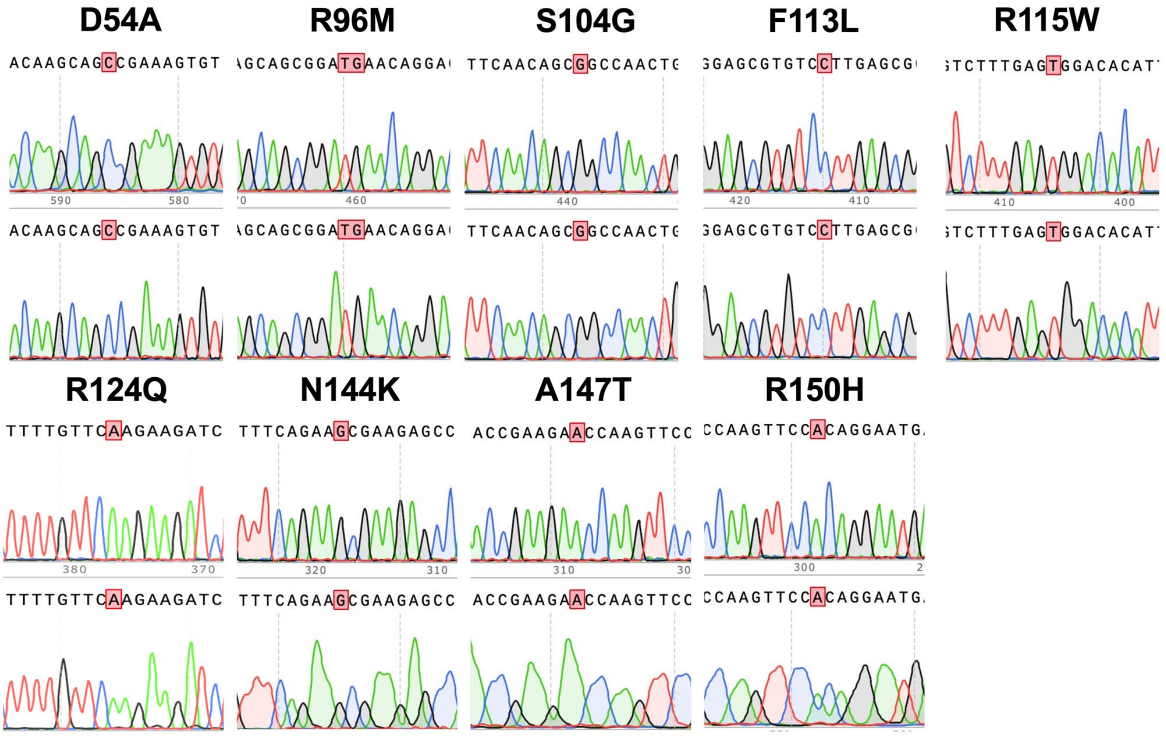

Supplemental Figure 3 Sanger sequencing traces of plasmids used in immunofluorescence analyses. Nine missense variants assayed in the immunofluorescence analysis. For each construct the forward and reverse reads are shown (top and bottom, respectively).

A

B

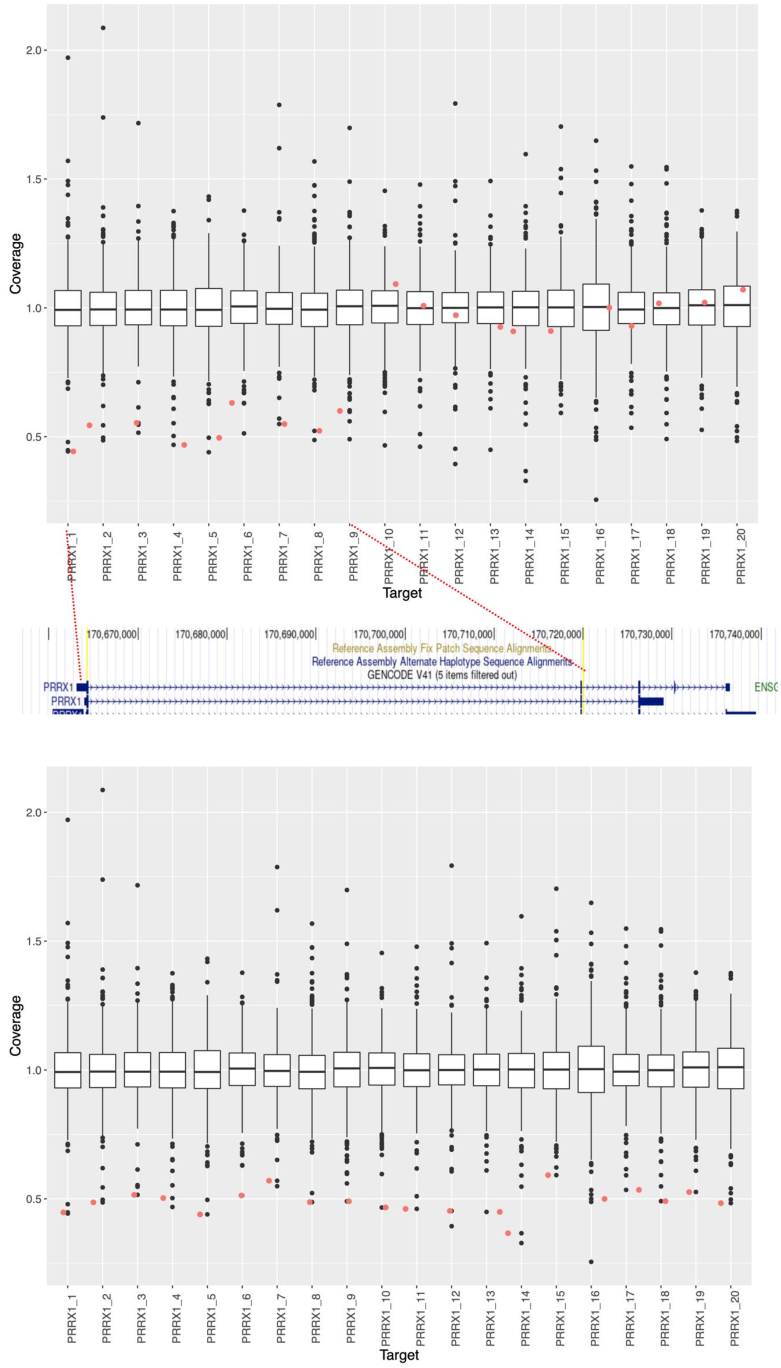

Supplemental Figure 4 Analysis of coverage of *PRRX1* in a screen of 479 unsolved craniosynostosis patients (A-B) Images show a boxplot of the normalized probe coverage for 20 non-overlapping regions in *PRRX1*. For each target, the horizontal centre line in the box marks the mean normalized coverage considering 479 samples, the top and bottom line of the box mark the upper (75th percentile; Q3) and lower (25th percentile; Q1) interquartile ranges (IQR). For each box plot, the vertical line extends to the maximum and minimum values (Q3 + 1.5*IQR and Q1 – 1.5*IQR, respectively). Any normalized probe coverage values above or below the maximum and minimum are annotated as a dot (outliers). (A) Partial deletion of *PRRX1* (sample II-2 from Family 14; red dots), resulting in reduced normalized coverage of probes 1–9 (below 0.6), corresponding to the deletion of exons 1–2 as shown by the yellow lines in the below UCSC screenshot (chr1: 170664249–170719963 [GRCh38]). (B) Whole gene deletion of *PRRX1* (sample II-1 from Family 15; red dots) shown by a drop in coverage (below 0.6) across all probes (spanning exons 1–5; chr1: 170664249–170736267 [GRCh38]).

A

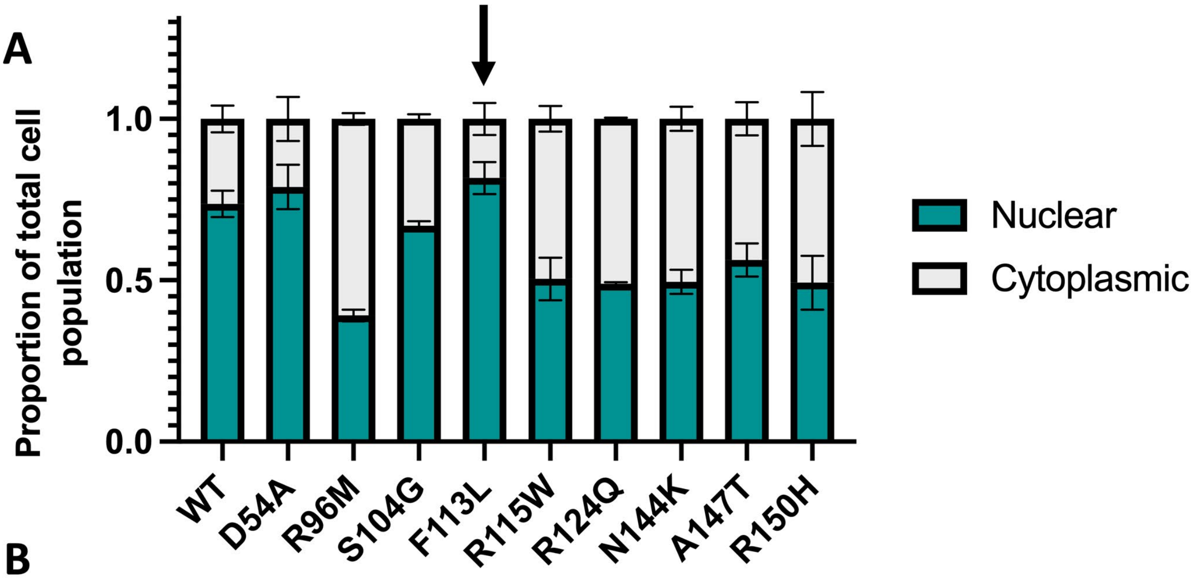

B

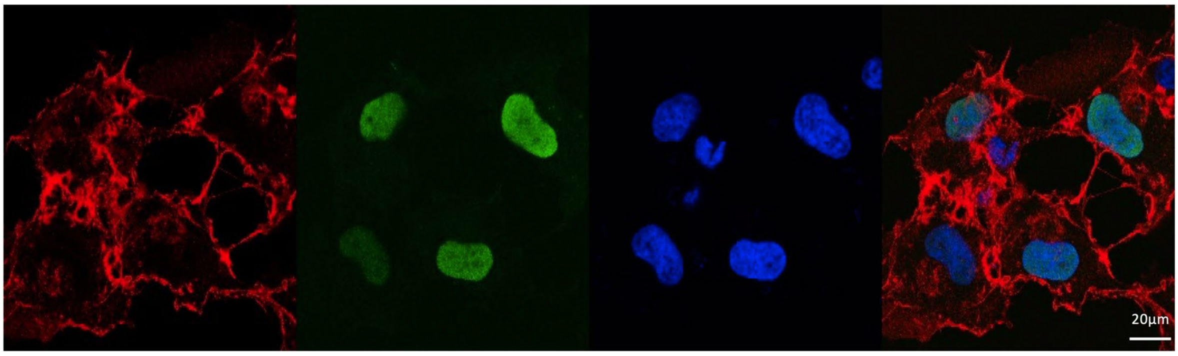

Supplemental Figure 5 Analysis of the p.(Phe113Leu) variant identified in a patient with agnathia-otocephaly. (A) Results from the ScanR analysis showed no significant difference between the proportion of nuclear cells in the F113L background compared to the wild-type protein. (B) Confocal microscopy showing four cells expressing the F113L construct with the cytoplasm stained in red (actin), PRRX1 stained in green, and the nucleus stained in blue.

REFERENCES

1. Koelling N, Bernkopf M, Calpena E, et al. Amplimap: a versatile tool to process and analyze targeted NGS data. *Bioinformatics*. Dec 15 2019;35(24):5349-5350. doi:10.1093/bioinformatics/btz582
2. Swain PK, CHen S, Wang Q-L, et al. Mutations in the Cone-Rod Homeobox Gene Are Associated with the Cone-Rod Dystrophy Photoreceptor Degeneration. *Neuron*. 1997;19:1329-1336.
3. Takagi M, Takahashi M, Ohtsu Y, et al. A novel mutaiton in HESX1 causes combined pituitary hormone deficiency withotu septo optic dysplasia phenotypes. *Endocrine Journal*. 2016;63(4):405-410.
4. Trochet D, Bourdeaut F, Janoueix-Lerosey I, et al. Germline Mutations of the Paired-Like Homeobox 2B (PHOX2B) Gene in Neuroblastoma. *Am J Hum Genet*. 2004;74:761-764.
5. Binder G, Renz A, Martinez A, et al. SHOX haploinsufficiency and Leri-Weill dyschondrosteosis: prevalence and growth failure in relation to mutation, sex, and degree of wrist deformity. *J Clin Endocrinol Metab*. Sep 2004;89(9):4403-8. doi:10.1210/jc.2004-0591
6. Dawson SJ, Palmer RD, Morris PJ, Latchman DS. Functional role of position 22 in the homeodomain of Brn-3 transcription factors. *Molecular Neuroscience*. 1998;9(10):2305-2309.
7. Wilkie A, Tang Z, Elanko N, et al. Functional haploinsufficiency of the human homeobox gene MSX2 causes defects in skull ossification. *Nature Genetics*. 2000;24:387-390.
8. Vieira TC, Dias da Silva MR, Cerutti JM, et al. Familial combined pituitary hormone deficiency due to a novel mutation R99Q in the hot spot region of Prophet of Pit-1 presenting as constitutional growth delay. *J Clin Endocrinol Metab*. Jan 2003;88(1):38-44. doi:10.1210/jc.2001-011872
9. Jorge AA, Souza SC, Nishi MY, et al. SHOX mutations in idiopathic short stature and Leri-Weill dyschondrosteosis: frequency and phenotypic variability. *Clin Endocrinol (Oxf)*. Jan 2007;66(1):130-5. doi:10.1111/j.1365-2265.2006.02698.x
10. Ross JL, Kowal K, Quigley CA, et al. The phenotype of short stature homeobox gene (SHOX) deficiency in childhood: contrasting children with Leri-Weill dyschondrosteosis and Turner syndrome. *J Pediatr*. Oct 2005;147(4):499-507. doi:10.1016/j.jpeds.2005.04.069
11. Goodman FR, Bacchelli C, Brady AF, et al. Novel HOXA13 mutations and the phenotypic spectrum of hand-foot-genital syndrome. *Am J Hum Genet*. Jul 2000;67(1):197-202. doi:10.1086/302961
12. Dreyer SD, Zhou G, Baldini A, et al. Mutations in LMX18 cause abnormal skeletal patterning and renal dysplasia in nail patella syndrome. *Nature Genetics*. 1998;19:47-50.
13. Bunyan DJ, Baker KR, Harvey JF, Thomas NS. Diagnostic screening identifies a wide range of mutations involving the SHOX gene, including a common 47.5 kb deletion 160 kb downstream with a variable phenotypic effect. *Am J Med Genet A*. Jun 2013;161A(6):1329-38. doi:10.1002/ajmg.a.35919
14. Pfaeffle RW, Savage JJ, Hunter CS, et al. Four novel mutations of the LHX3 gene cause combined pituitary hormone deficiencies with or without limited neck rotation. *J Clin Endocrinol Metab*. May 2007;92(5):1909-19. doi:10.1210/jc.2006-2177
15. Kelberman D, Turton JP, Woods KS, et al. Molecular analysis of novel PROP1 mutations associated with combined pituitary hormone deficiency (CPHD). *Clin Endocrinol (Oxf)*. Jan 2009;70(1):96-103. doi:10.1111/j.1365-2265.2008.03326.x
16. Barca-Tierno V, Aza-Carmona M, Barroso E, et al. Identification of a Gypsy SHOX mutation (p.A170P) in Leri-Weill dyschondrosteosis and Langer mesomelic dysplasia. *Eur J Hum Genet*. Dec 2011;19(12):1218-25. doi:10.1038/ejhg.2011.128
17. Sabherwal N, Schneider KU, Blaschke RJ, Marchini A, Rappold G. Impairment of SHOX nuclear localization as a cause for Leri-Weill syndrome. *J Cell Sci*. Jun 15 2004;117(Pt 14):3041-8. doi:10.1242/jcs.01152

18. Voronina VA, Kozhemyakina EA, O'Kernick CM, et al. Mutations in the human RAX homeobox gene in a patient with anophthalmia and sclerocornea. *Hum Mol Genet*. Feb 1 2004;13(3):315-22. doi:10.1093/hmg/ddh025
19. Schneider KU, Marchini A, Sabherwal N, et al. Alteration of DNA binding, dimerization, and nuclear translocation of SHOX homeodomain mutations identified in idiopathic short stature and Leri-Weill dyschondrosteosis. *Hum Mutat*. Jul 2005;26(1):44-52. doi:10.1002/humu.20187
20. ten Berge D, Brouwer A, Korving J, Martin JF, Meijlink F. Prx1 and Prx2 in skeletogenesis: roles in the craniofacial region, inner ear and limbs. *Development*. 1998;125:3831-3842.
21. Lu M-F, Cheng H-T, Lacy AR, et al. Paired-Related Homeobox Genes Cooperate in Handplate and Hindlimb Zeugopod Morphogenesis. *Developmental Biology*. 1999;205:145-157.
22. Chassaing N, Sorrentino S, Davis EE, et al. OTX2 mutations contribute to the otocephaly-dysgnathia complex. *J Med Genet*. Jun 2012;49(6):373-9. doi:10.1136/jmedgenet-2012-100892
23. Patat O, van Ravenswaaij-Arts CM, Tantau J, et al. Otocephaly-Dysgnathia Complex: Description of Four Cases and Confirmation of the Role of OTX2. *Mol Syndromol*. Sep 2013;4(6):302-5. doi:10.1159/000353727
